# Poster Session I - A122 VACCINATION UPTAKE IN IBD PATIENTS COMMENCING BIOLOGIC TREATMENT: A QUALITY IMPROVEMENT PROJECT

**DOI:** 10.1093/jcag/gwaf042.122

**Published:** 2026-02-13

**Authors:** S Asim, N Willett, J Jones

**Affiliations:** Internal Medicine, Dalhousie University Faculty of Medicine, Halifax, NS, Canada; Division of Digestive Care and Endoscopy, Nova Scotia Health Authority, Halifax, NS, Canada; Division of Digestive Care and Endoscopy, Nova Scotia Health Authority, Halifax, NS, Canada

## Abstract

**Background:**

Patients with inflammatory bowel disease (IBD) may be at an increased risk of vaccine-preventable diseases (VPD). Some of the VPDs include hepatitis A, hepatitis B, measles, mumps, and rubella. The Canadian Association of Gastroenterology (CAG) established clinical practice guidelines for immunization in patients with inflammatory bowel disease (IBD) in 2021 for live and inactivated vaccines. Maintaining appropriate vaccination status in patients with IBD helps optimize patient outcomes. However, the uptake of vaccination among IBD patients staring biologic therapy is variable.

**Aims:**

This QI project aimed to assess the quality of VPD management in IBD patients in the three months preceding the start of biologic therapy. Data was collected on the primary outcome of interest (rate of appropriate vaccination) based on the most recent Canadian clinical practice guidelines published by the Canadian Association of Gastroenterology. Data was also collected on vaccination status (documentation of childhood vaccination record or vaccine titres), vaccine administration (i.e. primary care physician or gastroenterologist), and type of advanced therapy.

**Methods:**

A retrospective chart review was performed using the local electronic medical record (EMR). Patients who were newly started on an advanced therapy between June 2023 and October 2024 were included in the study. Patients who were switched from a previous biologic or did not have sufficient clinic notes to corroborate the data were excluded. Preliminary analysis is descriptive in nature.

**Results:**

Data collection is in progress. Out of the 18 patients starting new biologic therapy, all patients were identified as non-immune against at least one VPD on the local EMR. However, there was insufficient record of whether patients received the proper vaccinations prior to starting their biologic. Only 1/18 patients had documented vaccinations for hepatitis A, hepatitis B, COVID, and streptococcus pneumonia administered by the patient’s gastroenterologist. We believe the low vaccination rates were likely because they were not captured by the EMR used by the gastroenterologist. Vaccines titres were checked by the gastroenterologist for 100 percent (18/18) patients prior to the initiation of an advanced therapy. The most common advanced therapy started in new patients was Entyvio (6/18 patients).

**Conclusions:**

In conclusion, the gastroenterologist checked vaccine titires for all the patients newly starting advanced therapies for their IBD. Titres were checked consistently in 100% of the patients. Only 1/18 patients were vaccinated against hepatitis A, B, COVID, and streptococcus pneuonia. Entyvio was the most common advanced therapy. Additional data will be collected from the EMR and from Panorama (provincial vaccination database), to verify which vaccines patients truly received prior to advanced therapies.

**Funding Agencies:**

None

